# Unscheduled Hospital Admission as a Prognostic Factor in the Oncologic Patient: A Retrospective Study

**DOI:** 10.7759/cureus.72029

**Published:** 2024-10-21

**Authors:** Carlos Maria Argerich, Canay Onder, Luis May, Javier Trujillano, Maria Nabal

**Affiliations:** 1 Medicine, Universitat de Lleida, Lleida, ESP; 2 Internal Medicine, University Hospitals Birmingham, Birmingham, GBR; 3 Palliative Care, Hospital Universitario Arnau de Vilanova, Lleida, ESP; 4 Intensive Care, Hospital Universitario Arnau de Vilanova, Lleida, ESP

**Keywords:** advancement in palliative care, cancer cachexia, cancer survival, emergency admission, oncological pain, palliative cancer, prognostic modeling, supportive and palliative care, survival analysis, unscheduled admission

## Abstract

Aims

This research aimed to determine the correlation between survival, symptoms, and unscheduled admission in oncologic patients. Furthermore, this study aimed to develop a prognostic model that helps clinicians establish the indication of intervention by palliative care teams.

Methodology

A retrospective study of patients’ digital clinical history registry was conducted to meet the two core objectives. The study population was patients with solid tumors undergoing unscheduled admissions to the oncology ward between January 1, 2018, and May 31, 2018. Demographic and clinical variables of those patients were analyzed. Specifically, the statistical analysis involved descriptive analysis, Kaplan-Meier curves, Log-Rank, and Chi-Squared Automatic Interaction Detection decision tree modeling.

Results

The results were obtained from 100 admissions of patients with an average age of 64. Of the patient cases examined, 67% (n = 67) were male. In 72% (n = 72) of the cases, patients presented with Stage IV tumors, and the most frequent primary tumor location among the admissions was lung, at 29% (n = 29). Intervention by the palliative care team occurred for 38% (n = 38) of patients. Mortality at 30, 90, 180, and 365 days was 34% (n = 34), 56% (n = 56), 71% (n = 71), and 78% (n = 78), respectively.

Hepatic metastasis was the main predictor of mortality at 30 days (65%, n = 13) and at 90 days (90%, n = 18). In the absence of hepatic metastasis, the presence of more than one symptom predicted a mortality rate of 70% at 30 days.

The main factor associated with mortality at 180 and 365 days was the tumor stage, with stage IV tumors having the highest mortality rate (84.7%, n = 61, and 90.3%, n = 65, respectively). Among the Stage IV population, the primary site shows a significant impact on survival, with colorectal/reproductive tumors being associated with decreased mortality.

Conclusion

Unscheduled admission is a negative prognostic factor in oncologic patients. An unscheduled admission can be expected to result in low survival in an oncologic patient, especially in those presenting with stage IV; involving non-colorectal/reproductive primaries; or presenting with pain, dyspnea, cachexia, or delirium.

## Introduction

Although there is a lack of accurate data regarding the survival of advanced cancer patients after unscheduled admissions [1,2], some studies have suggested that unscheduled admission can be a valuable prognostic factor [3-7], along with specific symptomatology presented by the patient at the time of admission, like pain, dyspnea, cachexia or delirium [2,4,8-11].

Unplanned admissions can be a turning point for cancer patients [7,12]. Prognostic factors such as unplanned admissions could aid clinicians with reevaluating individual goals of care, applying changes according to the patient’s best interests, shifting towards palliative care (PC), and avoiding therapeutic obstinacy [13].

Objectives

General Objective

The general objective was to ascertain the global survival after unscheduled admissions of oncologic patients. Furthermore, the study aimed to evaluate the impact of unscheduled admissions as a prognostic factor and indicator of intervention by the PC team.

Specific Objectives

The specific objectives were to determine the correlation between unscheduled admission and survival in oncologic patients; determine the impact of the presence of pain, dyspnea, cachexia, or delirium on the survival of the patient; and develop a prognostic model from the variables related to unscheduled admissions that will help clinicians to establish the indication of intervention by the PC team.

## Materials and methods

The design applied for this research was a retrospective study, with the clinical history available in the digital registry as a data source. Ethical clearance was given by the Institutional Ethics Committee, Hospital Universitario Arnau de Vilanova, Lleida (Approval No. CEIC-2354, dated October 29, 2020). The study population was patients with solid tumors who underwent an unscheduled admission to the oncology ward at the Hospital Universitario Arnau de Vilanova. Patients with solid tumors who underwent an unscheduled admission to the oncology ward between January 1, 2018, and May 31, 2018, regardless of the stage of illness, were included in this study. Admissions were considered unscheduled if patients were admitted for symptom control or additional evaluation that could not be completed in the outpatient clinic or emergency department. Hematologic patients and patients with scheduled admissions were excluded from the study.

Analysis was completed on the clinical histories of 100 unscheduled admissions, corresponding to approximately 40% of the unscheduled admissions to the oncology ward in the five months described in the inclusion criteria. The sample size was defined as 100 due to time limitations and patients were selected using simple random sampling.

The variables analyzed for this study included demographic factors (age, sex, and date of birth), characteristics regarding pathology (primary site, tumor stage, presence of metastasis), length of admission (calculated from date of admission and date of discharge), involvement of the PC team, presence of advanced care directives, symptomatology, and survival (calculated from date of discharge and date of death). Primary site data was separated by site into lung, colorectal, liver and pancreas, breast, otorhinolaryngologic, urologic, reproductive, central nervous system, musculoskeletal, unknown origin, and other. Presence of metastasis, separated by site into lung, hepatic, central nervous system, bone, peritoneal, lymph node, and other. 

We considered there was involvement of the PC team in the cases where a consult or referral to the PC team was done during admission, the patient was already being followed up by the PC team or the admission was done as shared care between Oncology and PC teams. Advanced care directives included the Plan Integrado de Intervencion Conjunta (Shared Integrated Intervention Plan, PIIC) and Documento de Voluntades Anticipadas (Advance Directives Document, DVA).

Symptomatology data refers specifically to the presence of pain, dyspnea, cachexia, or delirium. Symptomatology was only considered as present when the patient records indicated the symptom control as being a reason for admission or a new-onset symptom that could not be managed in the emergency department or required emergency admission for further investigation.

The data were analyzed using descriptive analysis of averages and proportions. We analyzed survival using Kaplan-Meier curves and Log-Rank (Mantel-Cox) test, with right-censoring at 365 days. Predictive modeling and segmentation of groups was done using Chi-Squared Automatic Interaction Detection (CHAID) decision trees. Likelihood-ratio, Chi-Squared test, and Bonferroni adjustment were applied to the CHAID decision tree modeling method. The software used was IBM SPSS Statistics for Windows, version 26 (IBM Corp., Armonk, NY, USA).

## Results

The data for 100 unscheduled admissions to the oncology ward was analyzed. The patients admitted were mostly male (67%, n = 67), with an average age of 64.9 years (SD = 12.4) and an average length of hospital stay of eight days (SD = 6). The PC team was involved in 38% (n = 38) of the total admissions (Table 1).

**Table 1:** Demographic variables PC: palliative care

Demographic variables		Count	%	Mean	Standard deviation
Sex	Male	67	67%	-	-
	Female	33	33%	-	-
Age		-	-	65	12
Involvement of PC team	No	62	62%	-	-
	Yes	38	38%	-	-
Length of hospital stay (days)		-	-	8	6
Outcome at 30 days	Deceased	34	34%	-	-
	Survived	66	66%	-	-
Survival of the deceased before 30 days		-	-	6	9
Outcome at 90 days	Deceased	56	56%	-	-
	Survived	44	44%	-	-
Survival of the deceased before 90 days		-	-	26	28
Outcome at 180 days	Deceased	71	71%	-	-
	Survived	29	29%	-	-
Survival of the deceased before 180 days		-	-	49	52
Outcome at 1 year	Deceased	78	78%	-	-
	Survived	22	22%	-	-
Survival of the deceased before 1 year		-	-	68	79

Most admissions were for patients with stage IV cancer (72%, n = 72), of which the most common primary site was pulmonary (29%, n = 29), followed by colorectal (18%, n = 18) and urologic (15%, n = 15). Metastasis was present in 66% (n = 66) of the patients and the most common metastasis site was the lung (25%, n = 25) followed by hepatic (20%, n = 20). Detailed information on primary sites, cancer stage, and metastasis can be found in Table 2.

**Table 2:** Oncologic variables

Oncologic Variables		Count	%
Primary site	Lung	29	29%
	Colorectal	18	18%
	Liver and pancreas	8	8%
	Breast	10	10%
	Otorhinolaryngologic	1	1%
	Urologic	15	15%
	Reproductive	5	5%
	Central nervous system	4	4%
	Musculoskeletal	0	0%
	Unknown origin	1	1%
	Other	9	9%
Tumor stage	I	3	3%
	II	8	8%
	III	17	17%
	IV	72	72%
Lung metastasis	Absent	75	75%
	Present	25	25%
Hepatic metastasis	Absent	80	80%
	Present	20	20%
Central nervous system metastasis	Absent	89	89%
	Present	11	11%
Bone metastasis	Absent	90	90%
	Present	10	10%
Peritoneal metastasis	Absent	83	83%
	Present	17	17%
Lymph node metastasis	Absent	82	82%
	Present	18	18%
Other metastasis	Absent	82	82%
	Present	18	18%
Overall presence of metastasis	Metastatic	66	66%
	Non-metastatic	34	34%

**Figure 1:**
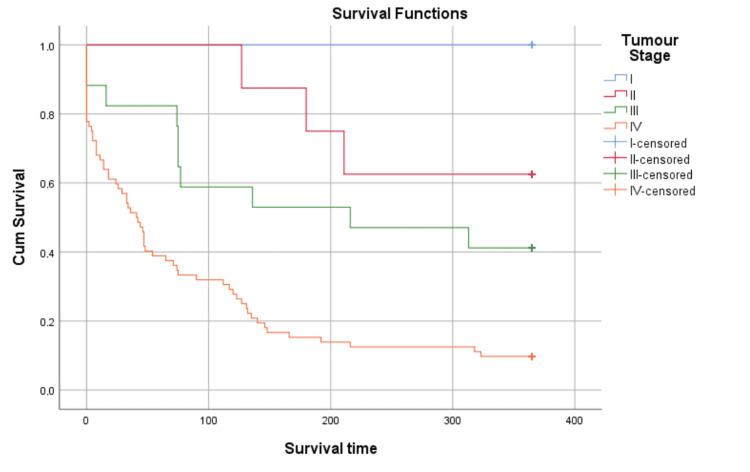
Kaplan-Meier survival, analysis stratified by tumor stage Right-censorship after 365 days. Censored n = 22

The impact of tumor stage on survival time is well illustrated by the Kaplan-Meier curve in Figure 1 and its significance is demonstrated in the statistical analysis in Table 3.

**Table 3:** Log-Rank (Mantel-Cox), tumor stage

Log-Rank (Mantel-Cox), tumor stage	Tumor stage	I		II		III		IV	
		Chi-Square	Sig.	Chi-Square	Sig.	Chi-Square	Sig.	Chi-Square	Sig.
Log-Rank (Mantel-Cox)	I	-	-	1.302	0.254	2.558	0.110	6.866	0.009
	II	1.302	0.254	-	-	1.290	0.256	10.978	0.001
	III	2.558	0.110	1.290	0.256	-	-	9.167	0.002
	IV	6.866	0.009	10.978	0.001	9.167	0.002	-	-

Symptomatology data (presence of pain, dyspnea, cachexia, or delirium as a reason for admission or a new-onset symptom) indicates the most common symptoms were pain (39%, n = 39) and dyspnea (31%, n = 31). In comparison, cachexia and delirium were rare at 2% (n = 2) and 7% (n = 7), respectively. 65% (n = 65) of the patients had one or more of these symptoms with the range being between two and zero symptoms and a mean of 0.79 (SD=0.671).

Advanced care directives (PIIC and DVA) were rarely found, with PIIC being present for 25% (n = 25) of the patients and only one instance of a DVA being completed. 

The outcomes and characteristics of each group at 30, 90, 180, and 365 days were examined (Tables 4, 5). Predictive modeling and segmentation of groups was done using CHAID decision trees, generating patient profiles and determining which variables significantly impact mortality.

**Table 4:** Correlates of survival at 30 and 90 days

Correlates of survival at 30 and 90 days		Outcome at 30 days				Outcome at 90 days			
		Deceased		Survived		Deceased		Survived	
		Count	Row N %	Count	Row N %	Count	Row N %	Count	Row N %
Primary cancer site (grouped)	Colorectal and reproductive	5	21.7%	18	78.3%	10	43.5%	13	56.5%
	All other primary sites	29	37.7%	48	62.3%	46	59.7%	31	40.3%
Tumor stage	I	0	0.0%	3	100.0%	0	0.0%	3	100.0%
	II	0	0.0%	8	100.0%	0	0.0%	8	100.0%
	III	3	17.6%	14	82.4%	7	41.2%	10	58.8%
	IV	31	43.1%	41	56.9%	49	68.1%	23	31.9%
Hepatic metastasis	No	21	26.3%	59	73.8%	38	47.5%	42	52.5%
	Yes	13	65.0%	7	35.0%	18	90.0%	2	10.0%
Overall presence of symptoms	Symptomatic	27	41.5%	38	58.5%	41	63.1%	24	36.9%
	Asymptomatic	7	20.0%	28	80.0%	15	42.9%	20	57.1%
Overall presence of metastasis	Metastatic	27	40.9%	39	59.1%	44	66.7%	22	33.3%
	Non-metastatic	7	20.6%	27	79.4%	12	35.3%	22	64.7%

**Table 5:** Correlates of survival at 180 days and 1 year

Correlates of survival at 180 days and 1 year		Outcome at 180 days				Outcome at 1 year			
		Deceased		Survived		Deceased		Survived	
		Count	Row N %	Count	Row N %	Count	Row N %	Count	Row N %
Primary cancer site (grouped)	Colorectal and reproductive	11	47.8%	12	52.2%	13	56.5%	10	43.5%
	All other primary sites	60	77.9%	17	22.1%	65	84.4%	12	15.6%
Tumor stage	I	0	0.0%	3	100.0%	0	0.0%	3	100.0%
	II	2	25.0%	6	75.0%	3	37.5%	5	62.5%
	III	8	47.1%	9	52.9%	10	58.8%	7	41.2%
	IV	61	84.7%	11	15.3%	65	90.3%	7	9.7%
Hepatic metastasis	No	52	65.0%	28	35.0%	59	73.8%	21	26.3%
	Yes	19	95.0%	1	5.0%	19	95.0%	1	5.0%
Overall presence of symptoms	Symptomatic	50	76.9%	15	23.1%	52	80.0%	13	20.0%
	Asymptomatic	21	60.0%	14	40.0%	26	74.3%	9	25.7%
Overall presence of metastasis	Metastatic	54	81.8%	12	18.2%	58	87.9%	8	12.1%
	Non-metastatic	17	50.0%	17	50.0%	20	58.8%	14	41.2%

Outcomes at 30 days

**Figure 2:**
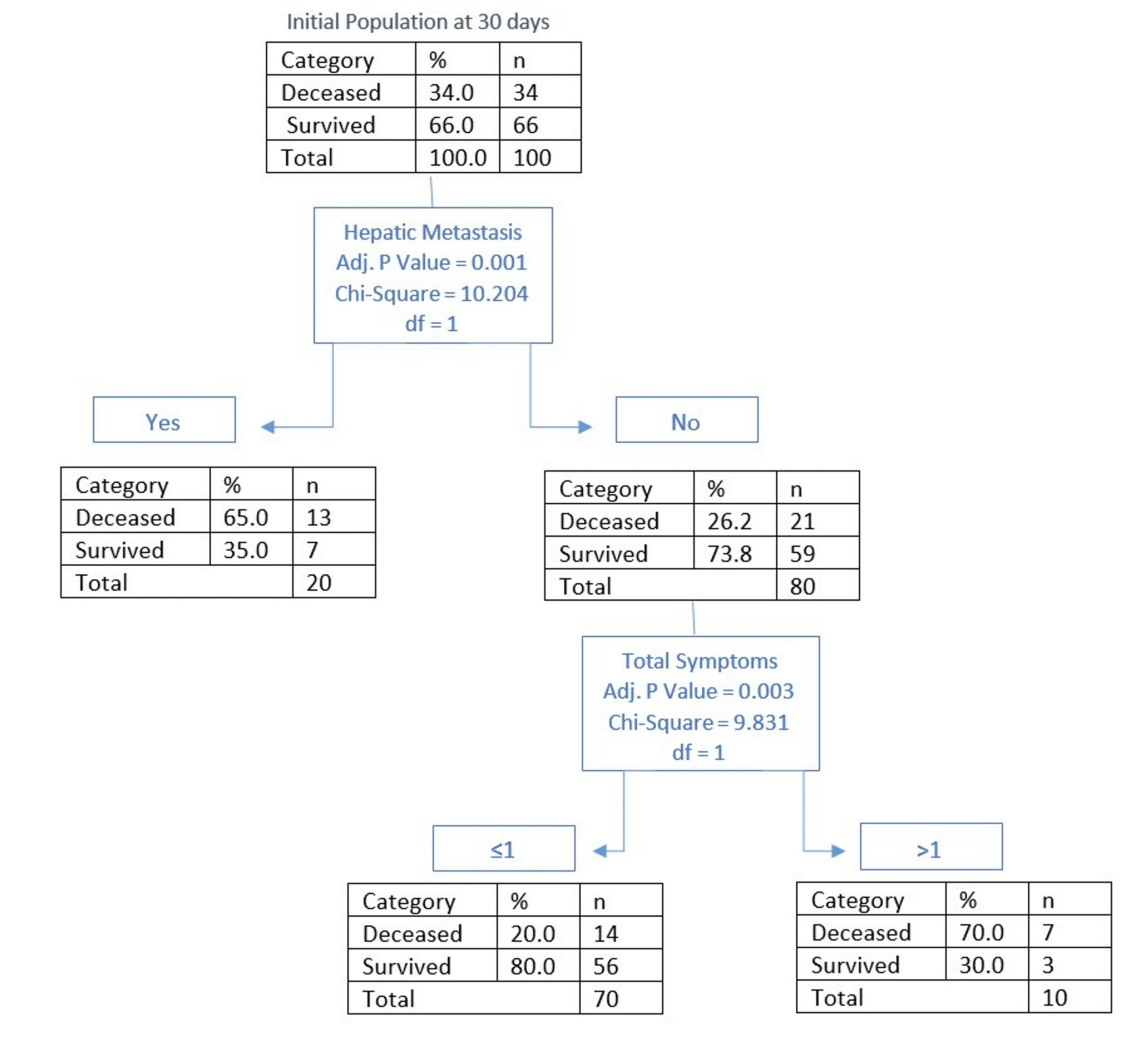
CHAID analysis of outcome at 30 days CHAID: Chi-Squared Automatic Interaction Detection

The mortality rate at 30 days was 34% (n = 34); survival of the deceased averaged at six days (SD=9). Based on CHAID analysis (Figure 2), the main predictor of mortality was found to be the presence of hepatic metastasis, where the presence correlated with a mortality of 65% (n = 13) versus 26.2% (n = 21) mortality in the absence group (p = 0.001). In the absence of hepatic metastasis, the presence of more than one symptom displayed a mortality rate of 70% (n = 7, p < 0.001). The population with one or less symptoms had a 20% (n = 14) mortality rate. Out of all deceased patients at 30 days, 70.5% (n = 24) had received specialized PC at the time of admission.

Outcomes at 90 days

**Figure 3:**
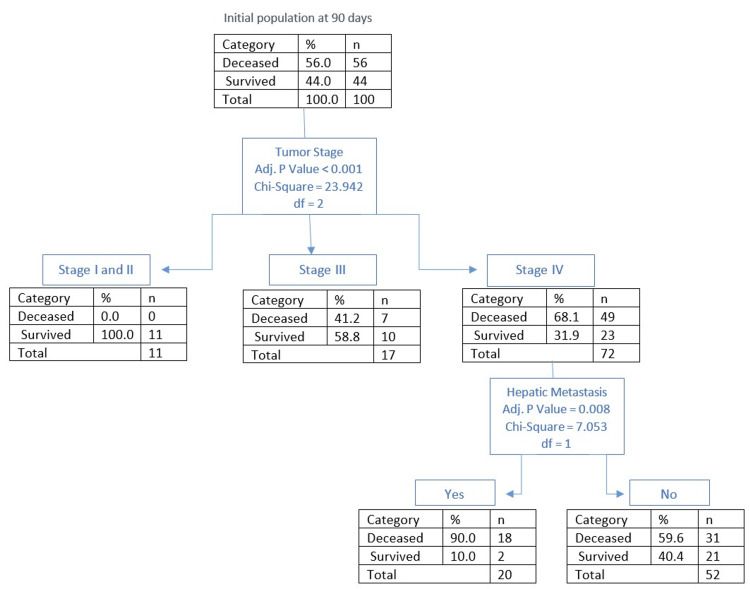
CHAID analysis of outcome at 90 days CHAID: Chi-Squared Automatic Interaction Detection

The mortality rate at 90 days was 56% (n = 56), with the average survival of the deceased being 26 days (SD=28). Based on CHAID analysis (Figure 3), the main contributing factors to mortality were tumor stage (68.1% mortality rate in stage IV, n = 49; 41.2% at stage III, n = 7; and 0% at stages I and II, n = 0; p < 0.001) and hepatic metastasis (90% mortality rate, n =18, p = 0.008). Out of all deceased patients at 90 days, 58.9% (n = 33) had received specialized PC at the time of admission.

Outcomes at 180 days

**Figure 4:**
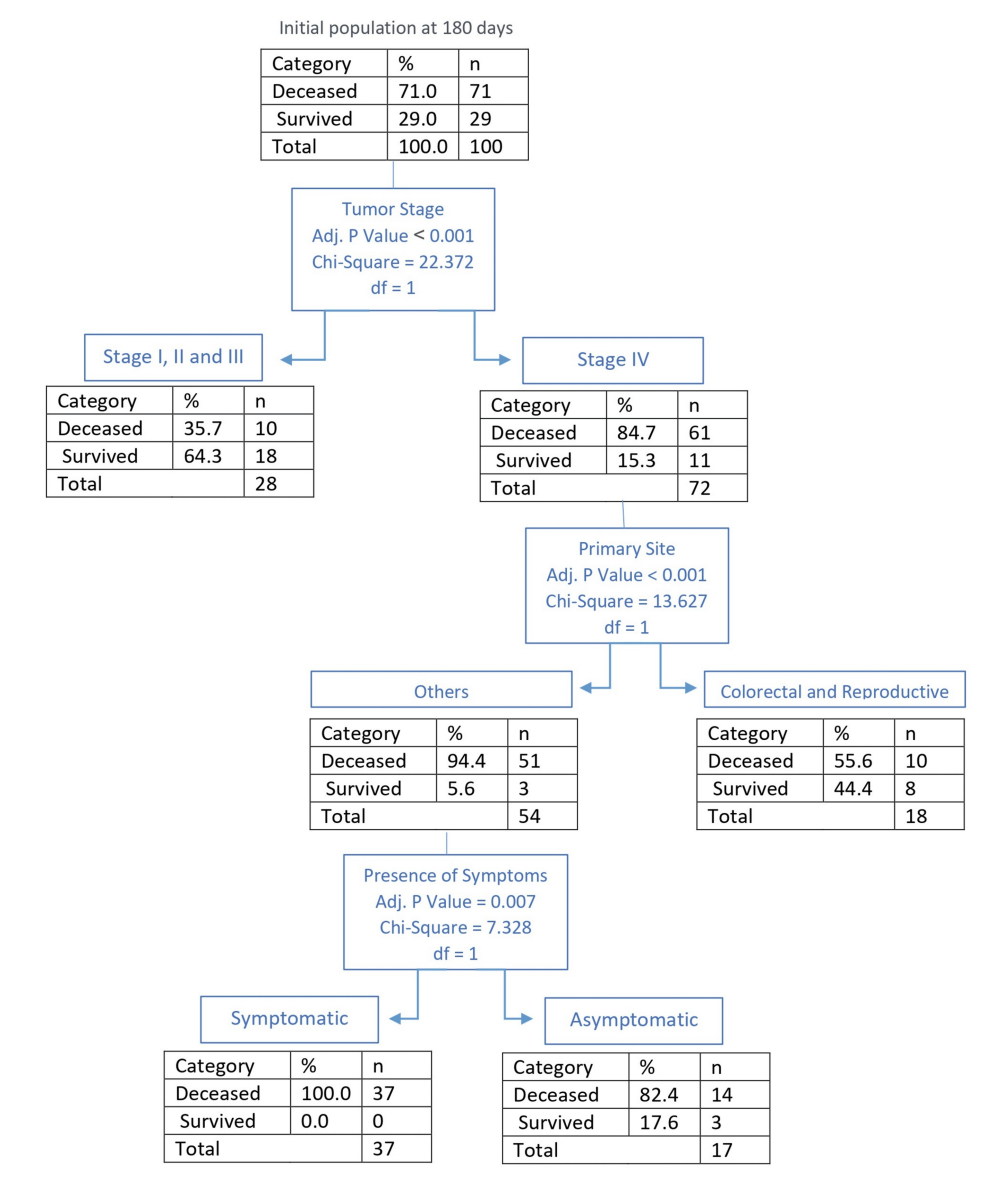
CHAID analysis of outcome at 180 days CHAID: Chi-Squared Automatic Interaction Detection

At 180 days, the observed mortality rate increased to 71% (n = 71), with the average survival of the deceased rising to 49 days (SD=52). Based on CHAID analysis (Figure 4), the main predictor of mortality was tumor stage, with stage IV displaying again a sharp increase in mortality with stage IV displaying a mortality rate of 84.7% (n = 61), and stage III, II, and I having a combined mortality rate of 35.7% (n = 10) (p < 0.001). Within the cohort of patients with stage IV tumors, CHAID analysis split the primary site categories into two significantly distinct groups. Those with colorectal and reproductive primaries had significantly better survival than patients with other primaries. In stage IV patients, there was a 55.6% mortality rate in colorectal or reproductive primaries (n = 10) versus a 94.4% mortality rate in all other primaries (n = 51) (p < 0.001). Within the other primary population group, those with any symptomatology presented a significant increase in mortality compared to those without symptoms (100% mortality rate with symptoms, n = 37, vs 82.4% mortality rate without symptoms, n = 14; p = 0.007). Out of all deceased patients at 180 days, 52.1% (n = 37) had received specialized PC at the time of admission.

Outcomes at 365 days

**Figure 5:**
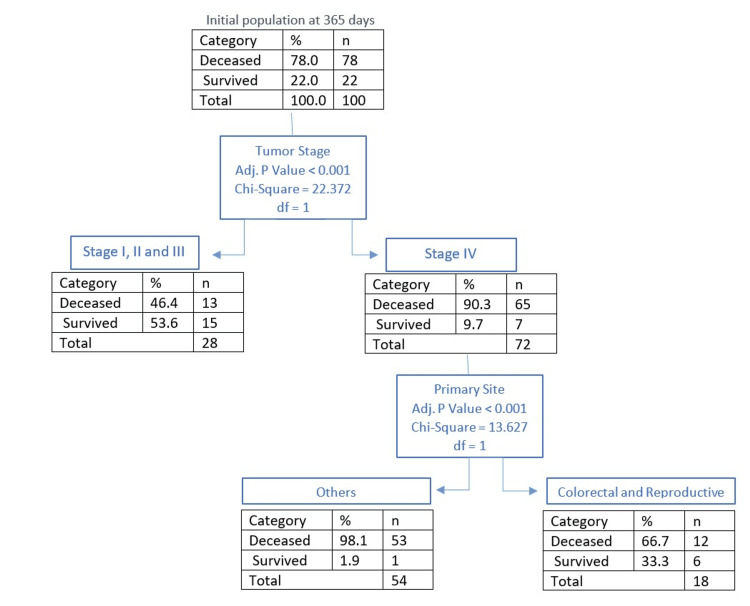
CHAID analysis of outcomes at 365 days CHAID: Chi-Squared Automatic Interaction Detection

At 365 days, the mortality rate reached 78% (n = 78), with the average survival of the deceased rising to 68 days (SD=79). Based on the CHAID analysis (Figure 5), the main predictor of mortality was tumor stage, with stage IV resulting in a mortality rate of 90.3% (n = 65) and stages I to III combined having a mortality of 46.5% (n = 13) (p < 0.001). Stage IV patients were again split into two significantly distinct groups according to their primary tumor site. Patients with colorectal or reproductive tumors showed a mortality rate of 66.7% (n = 12) and patients with all other types of primary tumor sites had a mortality rate of 98.1% (n = 53) (p < 0.001). Out of all deceased patients at 365 days, 47.4% (n = 37) had received specialized PC at the time of admission.

Based on the CHAID analysis performed across the different time periods, the variables with the most significant impact on survival were hepatic metastasis, tumor stage, primary site, and presence of symptoms.

## Discussion

This retrospective study aimed to analyze the patient population of oncologic patients who undergo unscheduled admissions to the oncology ward and shed light on their characteristics and outcomes, thereby allowing clinicians to understand the impact of such events better and plan in advance, according to the expected outcome and the patient’s decisions.

**Figure 6:**
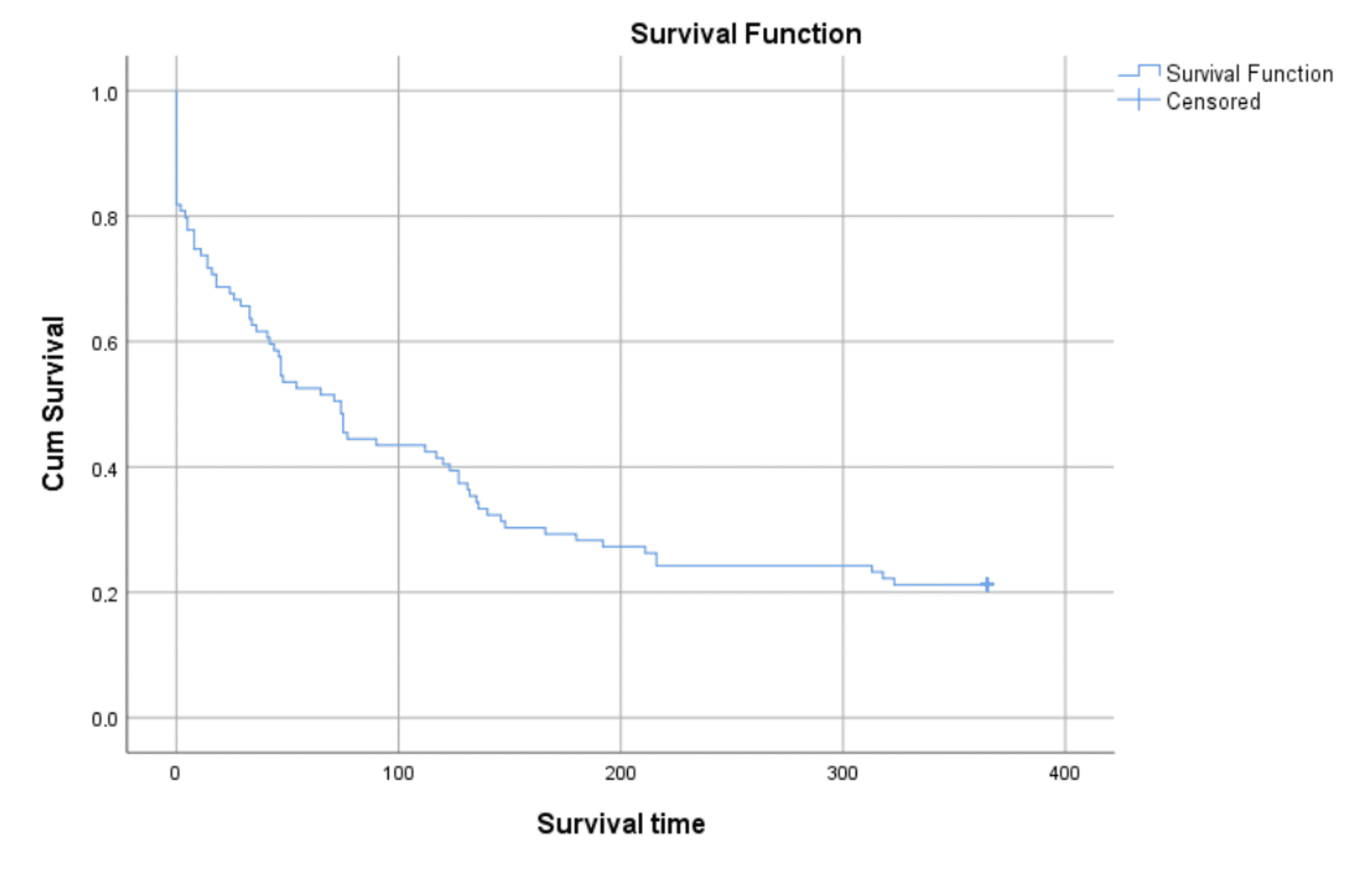
Kaplan-Meier survival analysis Right-censorship after 365 days. Censored n = 22

The analysis of the collected data shows that patients who undergo an unscheduled admission to the oncology ward have, even without prior consideration of their clinical characteristics, a very high short-term mortality rate (Figure 6). Prior research supports this assumption [3-7,14].

The results show that when the individual characteristics of the patients are analyzed with regard to their impact on survival, groups of patients with similar characteristics and survivability can be identified and their shared characteristics can be used as predictors.

**Figure 7:**
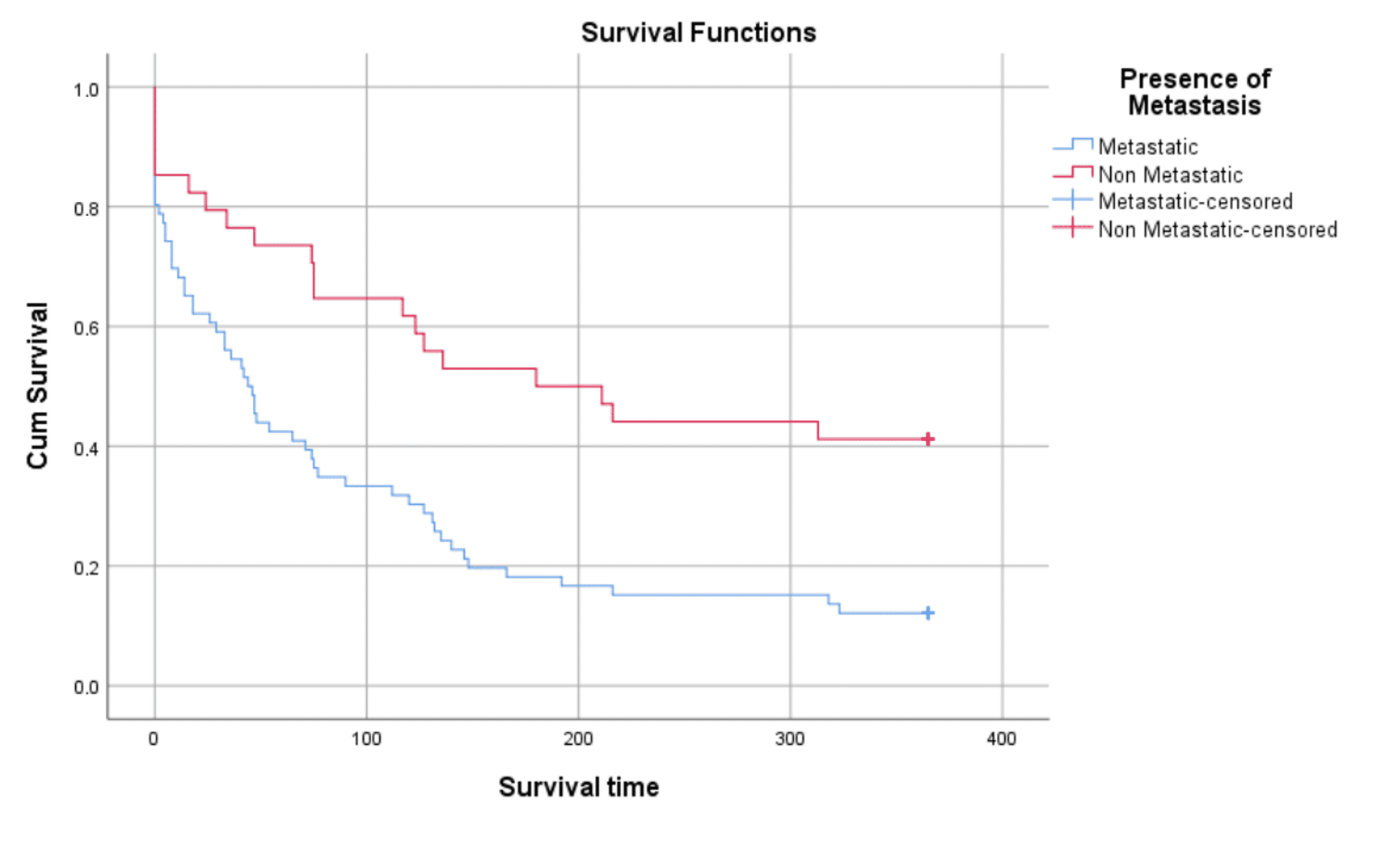
Kaplan-Meier survival analysis, stratified by presence of metastasis Right-censorship after 365 days. Censored n = 22 Log-Rank (Mantel-Cox): Chi-Square = 11.539, p = 0.001

**Figure 8:**
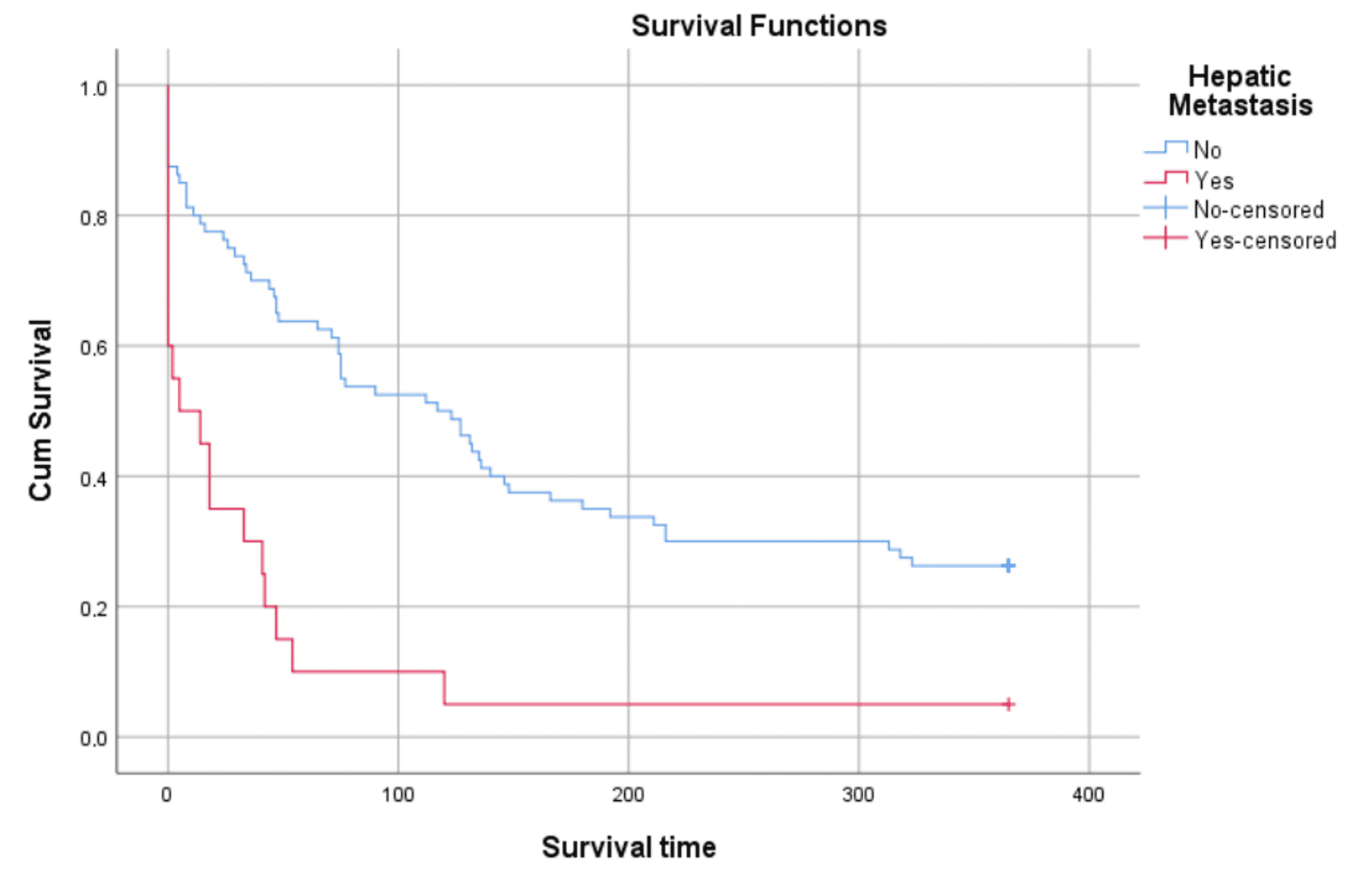
Kaplan-Meier survival analysis, stratified by presence of hepatic metastasis Right-censorship after 365 days. Censored n = 22 Log-Rank (Mantel-Cox): Chi-Square = 20.515, p < 0.001

The overall presence of metastasis is an important predictor of mortality (Figure 7), with the most important predictor being hepatic metastasis (Figure 8). As shown in Figure 2 and Figure 3, the presence of hepatic metastasis is correlated with a high mortality rate in a short period following admission. 

Within the population of stage IV cancer patients, different mortality rates can be predicted at 180 days and 365 days, depending on the primary tumor site. Colorectal and reproductive tumor sites correlate with improved survival when compared with other primary tumor site locations (Figures 4, 5). It is important to note that despite stage IV colorectal and reproductive primaries being predictors of improved survival compared to other stage IV primary sites, the presence of stage IV colorectal or reproductive primaries is still a factor that predicts a high mortality rate. Thus, its value as an indicator of improved survival needs to be considered within the context of stage IV cancer, and the high mortality rate this characteristic has been shown to predict on average (Figure 1, Table 3).

While no specific symptom has been highlighted as an individual predictor, the presence of any of the examined symptoms has been shown to correlate with poor prognosis in both the short and medium term (Figures 2, 4).

Due to the striking lack of completed advanced care directives in the clinical records, we cannot judge their impact or validity as predictor values.

The findings of this study share notable similarities with those of similar publications. Rocque et al. analyzed data from the years 2000 and 2010 which shows a similar mortality rate to that reported in our study after 365 days: 73.5% to 74.8% versus 78% (n = 78) respectively (Table 1) [4]. Rocque et al. also report that uncontrolled symptoms were the chief complaint in 70% to 66% of admissions, which is quite similar to the observed incidence (65%, n = 65) of the specific symptoms analyzed in this study [4].

Ostios-García et al. also followed similar objectives to those of this current research and concluded that unscheduled hospitalization is a sound criterion for PC referral [5]. In particular, Ostios-García et al. examined unscheduled hospitalization in the oncology service for over 1000 patients but considered only first admissions in their survival analysis, despite reporting a 58% rate of readmissions within the year following discharge [5]. They found a mortality of 69% after two years from the first admission compared to the current study’s report of 78% (n = 78) mortality after 365 days (Table 1). Readmissions have been shown to significantly decrease survival [15-17], which likely justifies the reported differences in mortality between the studies.

Considering the data obtained, which shows that the majority of these patients had an advanced illness, high short-term mortality, and important symptomatology, we consider that the recommendation for PC consultation at the moment of unscheduled admission was warranted in patients presenting with stage III and stage IV tumors or with specific symptoms. This assumption is supported by the consideration that, in the clinical setting, these patients would exceed the minimum criteria established by guidelines such as the National Comprehensive Cancer Network (NCCN) Palliative Care Guidelines [18,19], for specialized PC consultation in adult cancer patients. Further, these patients would meet the criteria for specialized PC intervention according to other PC screening tools, such as PALLIA-10 [20] or those proposed by Weissman and Meier [21] and Glare et al. [22], or recommendations for early intervention like those by Temel et al. [23]. The need for specialized PC is further supported by current literature, such as the research by Gemmel et al. [3], where medical records were compared against PC screening tools, and that comparison identified up to 91% of patients as needing PC in the six months before terminal admission.

The objective behind the recommendation for PC consultation at the moment of unscheduled admission would be to address end-of-life care for the large percentage of patients with poor short-term survival and ensure those patients benefit from early PC involvement in a proactive rather than reactive manner. Furthermore, the early involvement of the PC team would provide the necessary time and resources to appropriately discuss and implement living wills and advanced care directives. Early PC involvement would also allow these patients to make the best use of resources such as hospice care or home care units, for which most would qualify and benefit. Thereby, improving the care they receive and avoiding subsequent unnecessary hospitalizations or prolonged hospital stays.

Limitations

The main limitations of this study are its design and size. Being a retrospective study, sourcing information from digital clinical records poses limitations to the accuracy of our data that could better be addressed in a prospective study. The sample size used in this study and the unicentric design means that its conclusions may not be entirely accurate when applied to the general population, this could be solved with a larger sample size and a multicentric study design. The predictor models proposed in this study could also be tested prospectively to assess their accuracy and validity.

## Conclusions

We conclude that unscheduled admissions correlate with reduced survival in oncologic patients. We can conclude that unscheduled admissions, the overall presence of specific symptoms, and specific tumor characteristics (primary site, stage, and metastasis) can form useful prognostic models from which clinicians can predict outcomes at different survival times and base part of their clinical decisions accordingly. We conclude that palliative care consultation should be recommended in all patients undergoing unscheduled admissions to the oncology ward with stage III or stage IV tumors or presenting with pain, dyspnoea, delirium, or cachexia, regardless of tumor stage.
